# Developing early-career clinical educators – a qualitative study of a pedagogical internship track for junior doctors in Sweden

**DOI:** 10.1186/s12909-026-09549-1

**Published:** 2026-06-04

**Authors:** Thomas Ramo, Paul Pålsson, Hanna Wallberg, Helena Vallo Hult

**Affiliations:** 1 Department of Education, NU-Hospital Group, Trollhättan, Sweden; 2 Institute of Neuroscience and Physiology, Department of Clinical Neuroscience, Sahlgrenska Academy, University of Gothenburg, Gothenburg, Sweden; 3 School of Business, Economics and IT, Department of Informatics, University West, Trollhättan, Sweden

**Keywords:** Near-peer teaching, PAL, Peer-assisted learning, Health Professional Education, Faculty Development

## Abstract

**Background:**

Near-peer teaching (NPT) and structured near-peer teaching programs (NPTPs) have been recognized as valuable approaches in medical education, yet relatively few studies have explored programs designed specifically for junior doctors. In response to a local shortage of clinical supervisors and educators at a non-university teaching hospital in Western Sweden, a voluntary pedagogical faculty development program was launched in 2018 as part of the mandatory medical internship. The program engaged junior doctors with a documented interest in education to supervise medical students and peers. From the outset, the program was intended not only as a teaching initiative but also as an early form of faculty development, aiming to strengthen participants’ pedagogical skills and professional identity. This study aimed to explore how junior doctors participating in a pedagogical internship track experienced the development of their teaching skills and professional identity, as well as their experience of the program.

**Methods:**

We conducted a qualitative study of the pedagogical internship track using semi-structured interviews with ten former participants, enrolled between 2018 and 2024. Interviews explored their experiences of the program and its influence on subsequent professional roles. Data were analyzed using qualitative content analysis.

**Results:**

We identified three main themes: Theme 1, Building a pedagogical toolbox, highlighting how the participants acquired skills in simulation, feedback and how to orchestrate teaching events. Theme 2, Establishing professional legitimacy, with a focus on how the program gave them credibility as teachers and a platform to influence the teaching environment in the hospital. Finally, Theme 3, Becoming a clinical teacher, highlighting the development of an identity as a clinical teacher, growing confidence and how the participants took on new roles as clinical teachers.

**Conclusions:**

Findings from this study suggest that structured near-peer teaching programs for junior doctors strengthen teaching skills, enhance professional identity, and promote continued engagement in clinical education. By embedding such programs within internship training, hospitals may not only support individual development but also foster a sustainable pedagogical culture.

**Supplementary Information:**

The online version contains supplementary material available at 10.1186/s12909-026-09549-1.

## Introduction

Peer-assisted learning (PAL) and near-peer teaching (NPT) have been gaining an increasing amount of attention and popularity as effective strategies in medical education, from undergraduate level to specialization training [1]. PAL is defined as when individuals belonging to the same social group help each other to learn by teaching themselves, even though they are not trained professional educators [2]. As for NPT, the widely agreed upon definition is a form of peer-education where the teacher is performing the same training as the student, but being at least one year ahead [3]. Near-peer teaching programs (NPTPs) have been acknowledged across various academic disciplines [4] and have been proven to benefit both learners and those in teaching roles. Reported benefits for the tutors include consolidation of subject knowledge and clinical skills, development of communication and feedback competencies, improved organizational and planning abilities, and an enhanced understanding of educational theory and pedagogy. In addition, participation in NPTPs appears to cultivate positive attitudes toward teaching and may encourage future engagement in medical education [5].

Quality clinical education is crucial for maintaining medical training [6]. Even though there is a high level of motivation to teach among doctors [7], many healthcare systems still face challenges in recruiting and supporting clinicians as teachers [8]. Reported barriers include a high level of clinical workload, lack of allocated time for teaching and lastly, the lack of formal pedagogical training, which seems to contribute to the ambivalent attitude toward teaching duties [9].

Following the increasing interest in near-peer teaching programs in medical education, there has been a growing body of literature exploring the perspectives of senior medical students being tutors [10, 11], including from Sweden [12]. Yet, the data on the experience of junior doctors enrolled in such NPTPs within clinical settings is limited to a few studies [13–15]. Moreover, faculty development tends to focus on residents [16, 17]. Interns, however, tend to work in different clinics within a hospital and are usually involved in teaching medical students and peers [18]. Despite this, little is known about how structured pedagogical initiatives during internship shape teaching skills, professional identity, and opportunities for educational engagement.

In this study, we aimed to explore: (1) the impact of participation in the pedagogical internship track on future career choices; (2) the ways in which former participants continued to engage in teaching and supervision; and (3) the concrete skills and competencies acquired through the track and how these were applied in participants' current or previous positions.

## Methods

### Study setting

Medical education in Sweden leading to an MD degree is provided at seven independent universities [19]. During the study period the curriculum consisted of five and a half years in medical school, followed by a mandatory, structured clinical internship ranging from a minimum of 18 months up to 24 months – Allmäntjänstgöring (AT) – before being authorized to apply for a medical license from the National Board of Health and Welfare. During this internship, the junior doctor served a minimum of 3 months within internal medicine and surgical specialties respectively (but a combined minimum of 9 months), 3 months in psychiatry and 6 months in primary care. Thus, AT served as a transitional phase between undergraduate education and residency [19, 20].

The study setting is a regional, non-university hospital in Western Sweden, affiliated with the University of Gothenburg, where a voluntary pedagogical track within the internship program was introduced in the fall of 2018, designed as an NPT program. The purpose of the program is to engage junior doctors with a documented interest in teaching and education to supervise medical students during their clinical rotations. It is managed and financed by the head program directors for the hospital group. From 2018 to 2022 four candidates started the program yearly. To meet demand for interns able to supervise medical students and a higher demand for trained simulation facilitators, the program was expanded in 2022. Since then, eight candidates with an interest in and merit for teaching have been accepted into the program yearly.

The pedagogical internship track includes formal coursework in pedagogical skills and feedback, mostly arranged by local faculty but the participants also attend clinical teacher training at the University of Gothenburg. Training in simulation design, facilitation and debriefing is provided by the simulation center in the hospital group. During the program the participants supervise both peers and medical students in clinical settings as well as in simulations. The first curriculum was developed by the head program directors. Over time the contents of the program have continuously been developed and revised by the participants in collaboration with the head program directors. In this way, the program incorporates elements of both peer-assisted learning and near-peer teaching, allowing participants to develop educational skills across different learner levels. In total the curriculum is two months, spread over the 1.5-year-long internship.

### Respondents and recruitment

All 17 doctors who had completed the pedagogical internship track since its launch in 2018 were invited to participate in the study. They had enrolled in the program between 2018 and 2024.

A total of ten former participants agreed to take part in the study, six females and four males. Participants ranged in age from 29 to 46 years, and all had graduated from Swedish medical programs. At the time of the interviews, they were working within various medical specialties, as summarized in Table 1.

**Table 1:** Respondent characteristics

VARIABLE	DESCRIPTION
Number of respondents	10
Age (years)	29–46
Sex	6 females, 4 males
Medical education	Swedish universities
Year of graduation	2018–2022
Enrolled in program	2018–2024
Current specialties	Anesthesiology & Intensive Care; Family Medicine; Infectious Disease; Pediatrics; Orthopedic Surgery; Gynecology & Obstetrics; Psychiatry; Emergency Medicine

Written and oral study information was provided to all respondents prior to the interviews, and all gave informed consent. The information included background to the study, study procedures, and details regarding the handling of personal data.

### Data collection

A semi-structured interview guide was developed by the authors to explore respondents’ demographic background, current clinical roles and teaching responsibilities, experiences from the pedagogical internship track (including strengths and challenges), and their perspectives on peer- and near-peer teaching. The interview guide was piloted among current participants and underwent minor revisions (Appendix 1).

All interviews were conducted by the first author between 17 October and 13 December 2024. Eight interviews were conducted online, via Microsoft Teams [21], while two were held in person at the hospital. All interviews were audio-recorded. Interviews were carried out in Swedish, following the guide in a conversational style, with probing questions used to deepen and clarify responses. The duration of interviews ranged from 28 to 58 min. Data saturation was deemed to have been reached when no new themes emerged in later interviews.

### Analysis

The interviews were transcribed verbatim. Only the first author and the transcriber had access to the recordings, which were deleted after transcription. Transcripts were pseudonymized prior to analysis.

Qualitative content analysis was performed in accordance with Graneheim and Lundman [22]. All authors initially read the transcripts several times to familiarize themselves with the material and ensure immersion in the data. Meaning units were identified, condensed, and coded. Codes were then grouped into subthemes and, through iterative discussions between the authors, further refined into overarching themes. Quotations have been translated from Swedish to English by the authors. Minor linguistic adjustments were made to improve readability while preserving core meaning.

We chose to share a summary of the thematic framework with participants to enhance credibility. They confirmed that the findings reflected their experiences.

## Results

Three prevailing themes were found in the qualitative analysis: Building a pedagogical toolbox, establishing professional legitimacy and becoming a clinical teacher. Each theme is described in detail below, with illustrative quotes from the interviews (see Table 2 for a summary of themes and subthemes).

**Table 2:** Themes, subthemes and example quotes

THEME	SUB-THEME	EXAMPLE QUOTE
Building a pedagogical toolbox	Acquiring formal and practical education on medical simulations and feedback methods is readily useful in clinical practice	“Both the basic pedagogical course and the simulation instruction course give you a lot of tools to use when supervising and it feels like you can put more thought into it, even if you have a stressful everyday life. I think you got a lot of tips and tricks on how to think and how to do things… and I think that you have a baseline of a pedagogical mindset during your workday.” (R1)
	Emphasizing feedback	“Well, I would say above all that I’ve become much better at giving feedback – really, much better. I would say that is the most clearly tangible thing I have improved after the program.” (R5)
	Orchestrating teaching events and activities on a logistical level	"What I have learned is a little bit about planning, how to plan and organize, partly a lecture and partly how to plan an educational day." (R6)
Establishing professional legitimacy	Possibility for influence	“The strength during this time was that I got to reflect and be involved in shaping my own internship. Since I participated, I could influence how the other intern doctors experienced these simulation days. I chose scenarios based on what I felt I needed to learn more about.” (R4)
	Pedagogical experience as career advantage	“It should really be said, I also think you will notice later when applying for jobs that it is a major merit, and everyone thinks, I mean, it sounds very good to know about pedagogy… people are very interested and curious, and it allows you to stand out in a way that is very advantageous.” (R5)
	Group credibility	“I think the strengths have also been that you are in a group, that you can develop, and you can get a bit of inspiration from each other.” (R7)
	Having a local contact network within the hospital or workplace	“You get a very good foundation if you are interested in pedagogy and leadership, especially if you stay within the same hospital. You have contacts, you know, like [name of chief of internship and head program director], you know who they are and can email them, and hopefully they remember your name. That alone makes a big difference, I think.” (R2)
Becoming a clinical teacher	Growing confidence as a clinical teacher	“Yes, I think it gives credibility in pedagogical matters.” (R3)
	Developing a culture of peer teaching and learning	“Being relatively close to each other academically and career-wise makes it easier to understand how the other person thinks and reasons; it can be harder if you have been a consultant for 30 years to sense the medical student’s needs.” (R9)
	Taking on new roles as clinical teachers	“I am solely responsible for [the medical students’] education Monday to Friday full days, which is very tough but also very fun. I have faced many challenges in this, but it feels reassuring to have had a lot of pedagogical training behind me.” (R4)

*R* Respondent

### Theme 1: Building a pedagogical toolbox

#### Acquiring formal and practical education on medical simulations and feedback methods is readily useful in clinical practice

The opportunity to obtain formal and practical education on how to conduct medical simulations, along with theories underlying clinical supervision and feedback, was depicted as highly beneficial and educational. Several participants expressed their appreciation of the fact that they took formal coursework required at residency level, which in turn equipped them with tools to engage in their teaching activities and, importantly, to adopt a pedagogical approach even outside formal teaching contexts.

“Both the basic pedagogical course and the simulation instructor course give you a lot of tools to use when supervising and it feels like you can put more thought into it, even if you have a stressful everyday life. I think you got a lot of tips and tricks on how to think and how to do things… and I think that you have a baseline of a pedagogical mindset during your workday” (Respondent 1).

*“Having read extensively and received education in pedagogy and clinical supervision facilitates my daily clinical work.”* (Respondent 10).

Overall, receiving formal and practical training in medical simulation, leadership and feedback was perceived as a positive aspect of the pedagogical internship track, enhancing the participants' teaching abilities not only in formal teaching assignments but also in everyday clinical practice.

### Emphasizing feedback

Participation in the pedagogical internship track and the associated teaching assignments required the interns to regularly provide feedback to medical students and colleagues. Respondents described that this repeated practice not only improved their ability to deliver constructive feedback but also made them more available to receive feedback themselves. Several highlighted that they had become substantially more effective and confident in using feedback as a pedagogical tool.

*“ Well, I would say above all that I’ve become much better at giving feedback – really, much better. I would say that is the most clearly tangible thing I have improved after the program.”* (Respondent 5).

*"If you practice how to give feedback, you also understand how to receive feedback when you need it, and then you make yourself more available. I feel that you learn so much more if you open yourself up to feedback"* (Respondent 3).

Developing feedback skills was described as a highly important and empowering aspect of the pedagogical internship track, improving the participants' openness to receiving feedback in their own careers and future roles as resident doctors and clinical educators.

### Orchestrating teaching events and activities on a logistical level

Having the responsibility for different teaching activities within the hospital incentivized the participants to develop their ability to coordinate different logistical components, including booking lecture halls and finding suitable patients for the medical students to talk to.

*"What I have learned is a little bit about planning, how to plan and organize, partly a lecture and partly how to plan an educational day."* (Respondent 6).

* “It was about booking it (student room) and then going around looking for these patients myself”* (Respondent 2).

Nevertheless, several respondents hinted at the fact that these secondary tasks were excessive and time-consuming.

*"In our case there was so much booking of student rooms and booking, i.e. administration all around."* (Respondent 3).

Taken together, taking responsibility for the organization of different teaching activities enabled participants to develop valuable logistical skills, essential to work as clinical supervisors, although some of these tasks were perceived as time-consuming and administratively burdensome, with insufficient administrative support.

### Theme 2: Establishing professional legitimacy

#### Possibility for influence

Respondents emphasized the value of shaping both their own learning and the educational environment for their colleagues. Acting as pedagogical intern doctors allowed them to influence what and how other junior doctors learned, for example, by selecting scenarios and planning simulation days. This responsibility fostered engagement, ownership, and a sense of professional legitimacy, as they contributed directly to shaping the internship experience for their peers.

*“The strength during this time was that I got to reflect and be involved in shaping my own internship. Since I participated, I could influence how the other intern doctors experienced these simulation days. I chose scenarios based on what I felt I needed to learn more about.”* (Respondent 4).

Having the possibility to influence both personal learning and the learning conditions of colleagues enhanced participants’ professional agency and legitimacy, highlighting their role as active contributors to the broader educational environment within the medical internship.

### Pedagogical experience as career advantage

Participants described pedagogical experience gained during the pedagogical internship track as an asset for their future careers. Being involved in teaching activities and formal pedagogical training was perceived to enhance their professional profile. Several participants highlighted that this experience gave them credibility in pedagogical contexts and was seen as an advantage when applying for residency positions.

*“It should really be said, I also think you will notice later when applying for jobs that it is a major merit, and everyone thinks, I mean, it sounds very good to know about pedagogy… people are very interested and curious, and it allows you to stand out in a way that is very advantageous”* (Respondent 5).

* “It is definitely seen as a merit when applying for jobs…”* (Respondent 4).

In summary, pedagogical experience during the medical internship was seen as a career-enhancing advantage, providing professional credibility in teaching situations, perceived as a way to help respondents distinguish themselves from peers in the job market.

### Group credibility

Participants highlighted that being part of a pedagogical group contributed to their credibility and legitimacy within the hospital setting and among peers. Belonging to a designated and structured team, supported by experienced supervisors being their employer, provided a “stamp of approval” that made it easier to initiate projects and be taken seriously by colleagues. Being in a group also facilitated mutual learning, inspiration, and professional development, as members could support and motivate each other.

*“I mean, our strength was that we were pedagogical and had, like, [name of chief of internship and head program director] behind us, and I think that is important. I think it is harder to just randomly start a project without some form of approval from someone.”* (Respondent 1).

* “I think the strengths have also been that you are in a group, that you can develop, and you can get a bit of inspiration from each other.”* (Respondent 7).

Overall, being part of a pedagogical group enhanced participants’ credibility and confidence, while also providing opportunities for mutual support and professional growth.

### Having a local contact network within the hospital or workplace

The respondents, being former participants, emphasized the value of building a local professional network within the hospital. Engaging in pedagogical activities allowed them to establish connections with other supervisors and colleagues, providing both guidance and potential future support. Knowing key individuals personally, and having the opportunity to interact with them, were perceived as enhancing professional confidence and easing future collaboration. Establishing these contacts was also described as enjoyable and educational, contributing to both personal and professional development.

*“You get a very good foundation if you are interested in pedagogy and leadership, especially if you stay within the same hospital. You have contacts, you know, like [name of chief of internship and head program director], you know who they are and can email them, and hopefully they remember your name. That alone makes a big difference, I think”* (Respondent 2).

* “You make contacts in this way, and it was very nice, fun, and educational.”* (Respondent 4).

Thus, participating in pedagogical activities provided participants with a valuable local contact network, enhancing both professional confidence and opportunities for future collaboration within the hospital.

### Theme 3: Becoming a clinical teacher

#### Growing confidence as a clinical teacher

Respondents described how involvement in the pedagogical internship track program increased their confidence and sense of credibility as clinical teachers. Being actively engaged in teaching tasks allowed them to gain experience, build expertise, and feel prepared for future pedagogical responsibilities. Several participants emphasized that early involvement provided both practical skills and legitimacy in educational matters, making them feel more capable of taking on formal teaching roles.

*“I probably wouldn’t have felt ready to become a program director (for current intern doctors) if I hadn’t already been somewhat involved myself as an intern doctor in these matters.”* (Respondent 9).

* “I think it gives credibility in pedagogical matters.”* (Respondent 3).

Overall, participation in pedagogical activities during the internship seems to have fostered confidence and credibility as clinical teachers, supporting participants’ readiness to assume teaching responsibilities in the future.

### Developing a culture of peer teaching and learning

Participants emphasized that the near-peer structure of the pedagogical internship track facilitated effective teaching and learning among junior doctors. Working closely with colleagues at similar stages of training made it easier to understand each other’s perspectives, anticipate learning needs, and provide relevant, relatable feedback. This near-peer approach fostered a supportive and psychologically safe learning environment, enhancing both engagement and learning outcomes.

*“Being relatively close to each other academically and career-wise makes it easier to understand how the other person thinks and reasons; it can be harder if you have been a consultant for 30 years to sense the medical student’s needs”* (Respondent 9).

* “There is a higher level of openness when doing peer-assisted learning; people are more humble about what they know and don’t know, and being in a safe group makes learning much more effective. You can see it with the students—those at the same level, friends, feeling safe to admit ‘I might not know this, you know it better,’ it creates a much better working environment”* (Respondent 4).

Ultimately, this near-peer teaching was seen as a way to strengthen both learning and professional collaboration, empowering participants to shape a supportive and responsive educational environment for their colleagues.

### Taking on new roles as clinical teachers

As former participants, the respondents emphasized that the pedagogical skills gained during the pedagogical internship track had a lasting impact, enabling them to assume diverse educational roles after the program. In their subsequent positions as residents, they became actively involved in supervising medical students, mentoring new interns, and, in some cases, even taking on leadership roles such as program directors. Although these responsibilities were often demanding, they felt well-prepared and reassured by the pedagogical foundation established during their internship.

*“I am solely responsible for [the medical students’] education Monday to Friday full days, which is very tough but also very fun. I have faced many challenges in this, but it feels reassuring to have had a lot of pedagogical training behind me.”* (Respondent 4).

* “I have had the clinical consultation skills course at the clinic, and with the new medical program (in Sweden), I have now had the same group two semesters in a row.”* (Respondent 3).

These accounts illustrate how the near-peer teaching experiences during the internship were perceived to create long-lasting pedagogical engagement, with participants continuing to contribute actively as clinical teachers in their new positions as residents.

## Discussion

The aim of this study was to explore how junior doctors participating in a pedagogical internship track experienced the development of their teaching skills and professional identity, as well as how they perceived the program to have influenced their professional development. Our findings demonstrate that participation in the pedagogical internship track supported the development of a pedagogical toolbox, improved feedback skills, fostered a sense of professional legitimacy during their time as junior doctors, and lastly contributed to the development of an identity of clinical educators, which participants carried into their subsequent roles as resident doctors.

Several studies have documented the benefits of NPTPs in medical education, although most have focused on the undergraduate level [1]. While some reports describe advantages for junior doctors, such as enhanced teaching ability, professional development, and improved knowledge and skills, few have addressed the internship period immediately following graduation. Our findings extend the existing literature by showing that structured NPTPs during internship can support both pedagogical skills and identity formation. This was particularly evident in themes one and three of our study, where participants highlighted feedback as a core competence and described a growing confidence as clinical teachers. The pedagogical internship track can also be understood as a faculty development initiative, aligning with several of the key features described by Steinert and colleagues [5], including experiential learning, relevant and applicable content, opportunities for feedback, intentional community building, and strong institutional support. Importantly, our findings resonate with established faculty development models that emphasize not only the acquisition of teaching skills and confidence but also professional identity formation, community building, and sustained engagement in educational roles.

The outcomes illustrate well-documented benefits of faculty development programs, including enhanced credibility, legitimacy, and local networks within the workplace. We interpret these findings as evidence that the pedagogical internship track contributed to the development of a collaborative learning environment within the hospital, characterized by a strong sense of belonging, which facilitated future teaching initiatives as participants advanced to residency. O’Sullivan and Irby [23] distinguish between communities of practice that develop among program participants and those that manifest within the workplace. While the former is perhaps expected, our findings suggest that the pedagogical internship track also had a broader institutional impact. As interns rotate through multiple hospital departments, they can disseminate a “pedagogical culture,” as one respondent expressed it, thereby normalizing teaching and learning as valued professional activities. Such a culture may help strengthen the educational mission of the hospital.

Medical educators are essential for sustaining the medical profession, as they play a central role in teaching medical students, junior doctors, residents, and in supporting continuing professional development [24]. Although many physicians express strong motivation to teach, multiple challenges, including increasing clinical workload and limited protected time for education, restrict their ability to engage in teaching. Estimates suggest that as few as 10% of clinicians can teach within their allocated time [25]. One way to address these discrepancies is through faculty development initiatives for early-career doctors. Programs such as a pedagogical internship track strengthen participants’ professional profiles and encourage them to assume new roles as clinical educators, as illustrated by theme three of our study. 

### Implications for practice

Our findings suggest that structured near-peer teaching programs for junior doctors can have several practical implications. Although this program was developed within the Swedish internship system, the core elements of structured teaching opportunities, pedagogical training, and institutional support are transferable to other contexts and may help address the global shortage of clinical teachers [6]. By equipping junior doctors with skills and confidence, such programs not only strengthen their professional development but also enhance the supervision and feedback provided to medical students and peers, thereby improving the quality of clinical learning environments. Importantly, as emphasized in previous research [15], sustained success requires strong institutional and organizational support. Several of our respondents likewise identified the need to optimize the administrative support system for participants as an area for improvement. In addition, initiatives such as the pedagogical faculty development program described in this study may be particularly valuable in non-university hospitals, where pedagogical infrastructures are often less developed [26]. Embedding early faculty development tracks in these settings could help build a stronger teaching culture and ensure that clinical education remains a prioritized mission. Integrating programs like a pedagogical internship track into postgraduate training, while ensuring adequate organizational infrastructure, thus represents a sustainable strategy for healthcare systems that aligns workforce development with the educational mission of hospitals.

### Strengths and limitations

A strength of this qualitative study is the inclusion of former participants from the pedagogical internship track across multiple starting groups since its introduction in 2018, providing a comprehensive representation of the program over time. Furthermore, the use of respondent validation strengthened the credibility of the findings, as participants confirmed the accuracy of the thematic interpretation.

A potential limitation of this study is that several of the authors are involved in the pedagogical internship track, with preunderstandings and investment in its success, potentially influencing the results, reducing trustworthiness. However, to mitigate the potential bias our research team was deliberately set up to include both insider and outsider perspectives. The interviews were conducted by a peer to the respondents, himself a participant in the program, allowing access to shared tacit knowledge with the respondents. Two of the authors are program directors, responsible for the design and management of the program, providing institutional knowledge to the analysis. Finally, the last author is a senior non-physician educational researcher with no previous involvement in the program, allowing an outsider perspective and a position to challenge assumptions. We find the insider–outsider dynamic in the researcher team a strength for the validity of our results.

Additional limitations include the focus on former participants only, excluding perspectives from current interns or other professional groups. Furthermore, as the interviews were conducted in Swedish and later translated into English, some nuances in meaning may have been lost. While the qualitative approach enabled an in-depth and context-rich understanding, future studies could expand on these findings by including multiple institutions that have similar initiatives, other professional groups, or complementary quantitative approaches.

## Conclusion

Near-peer teaching and its application as near-peer teaching programs have long been recognized within the field of medical education although relatively few studies have explored and evaluated such programs intended for junior doctors. In line with recommendations from the field, this study evaluated an ongoing near-peer teaching program (NPTP) for junior doctors in Western Sweden. Our findings demonstrate that the program supported the development of pedagogical skills, fostered professional legitimacy, and contributed to the formation of a clinical teacher identity among participants. These outcomes suggest that structured NPTPs can serve as an early form of faculty development, encouraging junior doctors to take on educational roles from the beginning of their careers. By strengthening teaching capacity during internship, programs like the one discussed in this article may help address the ongoing shortage of clinical educators and foster a stronger pedagogical culture within healthcare. Future research could explore long-term impacts on both participants and the students they supervise, as well as the transferability of this model to other settings.

## Supplementary Information

Supplementary Material 1.

## Data Availability

The dataset from the study is available from the corresponding author upon reasonable request.
