# Supplementary material for: Developing early-career clinical educators – a qualitative study of a pedagogical internship track for junior doctors in Sweden
Source: BMC Med Educ. 2026 Jun 4;26:910. doi: 10.1186/s12909-026-09549-1 (PMC13235182; doi:10.1186/s12909-026-09549-1)
Supplement: Supplementary file 1 — Supplementary Material 1. [file 12909_2026_9549_MOESM1_ESM.docx]

**Interview Guide: Pedagogical Internship Track**

**Project:** Exploring the experiences of former participants of the program.
**Format:** Semi-structured interview
**Duration:** Approximately 1 hour
**Setting:** Digital video call (Microsoft Teams) or in-person
**Interviewer:** Thomas Ramo
**Participants:** Former participants of the pedagogical internship track

**Key Research Questions**

- What impact did participation in pedagogical internship track have on future career choices?
- In what ways do former participants continue to engage in teaching and supervision?
- What concrete skills and competencies did participants acquire through pedagogical internship track, and how are these applied in their current or previous positions?
- How did participation in pedagogical internship track influence participants’ perspectives on their own learning?

**Structure and Interview Questions**

**Introduction and background**

- Could you please introduce yourself, including your medical degree, current role, and workplace?

**Motivations and entry**

- Why did you apply for pedagogical internship track? Did you have any prior experience or background in teaching or education?

**Experiences and reflections**

- What was your overall experience of participating in pedagogical internship track?
- What was your next career step after completing the pedagogical internship track?
- Have you continued to teach or supervise medical students or colleagues in your current position?
- Do you believe that participating in pedagogical internship track has influenced the roles you now hold?
- Has pedagogical internship track increased your interest in taking on educational or teaching responsibilities in the future?
- Has pedagogical internship track affected your opportunities to obtain educational assignments during your career?

**Skills and learning**

- What specific skills or competencies did you gain from your time in pedagogical internship track?
- Did participating in pedagogical internship track influence your own approach to learning?

**Program evaluation**

- In your view, what are the strengths and weaknesses of pedagogical internship track?
- What do you see as the strengths and weaknesses of the peer-assisted learning and near peer learning model?
- Is there anything else about pedagogical internship track that you would like to highlight that we have not yet discussed?

**Closing**

- Concluding remarks and thanks
